# Associations between vitamins intake and risk of cancer in United States adults: 2003 to 2016 national health and nutrition examination survey

**DOI:** 10.3389/fnut.2025.1561251

**Published:** 2025-04-02

**Authors:** Youfei Wen, Xiuzhen Yang, Yan Huang

**Affiliations:** ^1^Department of Gastroenterology, Guizhou Hospital of the First Affiliated Hospital of Sun Yat-sen University, Guiyang, China; ^2^Department of Gastroenterology, Gui’an Hospital of the Affiliated Hospital of Guizhou Medical University, Guiyang, China; ^3^Department of Gastroenterology, The Affiliated Hospital of Guizhou Medical University, Guiyang, China

**Keywords:** vitamins, cancer, niacin, vitamin A, NHANES

## Abstract

**Introduction:**

National Health and Nutrition Examination Survey (NHANES) is a cross-sectional survey that gathered information about people’s health and nutrition. The aim of this study is to investigate potential associations between vitamin intake and cancer risk using this database.

**Methods:**

The NHANES data set encompassed a wide range of variables, including vitamins, cancer, and others. Logistic regression models, restricted cubic splines (RCS) and subgroup analysis were used to test the potential link between vitamin consumption and cancer risk.

**Results:**

In total, 29, 138 individuals were included in this study, while 2,924 of them had a diagnosis of cancer. The odds of developing cancer were reduced for persons consuming the highest quartile of dietary niacin compared to those consuming the lowest quartile [odds ratio (OR) = 0.78, 95% confidence range = 0.65, 0.95, *p* = 0.015]. However, after adjusting for all confounding factors, as the intake of vitamin A gradually increased, the risk of tumor occurrence correspondingly increased (OR = 1.38, 95% CI 1.13, 1.69, *p* = 0.002). Subgroup analysis and RCS models showed similar results. Only when the intake of folic acid is 267–367 mcg, folic acid is positively correlated with the risk of tumors. Vitamins E, B1, B2, B6, B12, C, K, alpha-carotene and beta-carotene were not associated with the risk of tumor development.

**Conclusion:**

Vitamin A intake is positively correlated with the occurrence of tumors, while niacin intake is negatively correlated with the incidence of tumors. Further longitudinal studies are needed to verify these findings.

## Introduction

Public health is facing a significant and growing challenge in the form of cancer, particularly in Western societies that are getting older. As of 2019, cancer was the second leading cause of death in Western Europe, accounting for 30 % of all deaths that occurred in that region (1). Cancer is typically characterized as a collection of cells that can proliferate uncontrollably and sustain themselves by relocating to a more favorable environment for their growth, referred to as a malignant tumor or cancer (2). Physiological homeostasis is widely recognized to deteriorate with advancing age. Dietary and lifestyle decisions profoundly influence the aging process (3). There are many different components of food that can have an effect on human health. Some examples of these components include polyphenols, minerals, and vitamins. Due to the fact that these components possess anti-inflammatory and antioxidant properties, they have the ability to slow down the process of cellular senescence, which in turn prevents the development and progression of cancer (4, 5).

Many physiological processes rely on vitamins, and these nutrients also have far-reaching effects on people’s general health. Vitamins’ potential to ward off cancer has garnered considerable interest in the last several decades (6). Vitamins control cell growth, differentiation, energy metabolism, and cell death in their many manifestations (7, 8). It controls angiogenesis, immune response, and other basic cancer-related processes under normal physiological conditions as well. Some forms of cancer have been associated with vitamin deficiencies as well (9). Cancers were all lowered in incidence when B vitamins were taken. On the other hand, certain B vitamin supplements may raise cancer risk and have other negative side effects (10). A vitamin B12 deficiency is more likely to occur in older patients and those whose cancer is in its early stages (11). Eating foods rich in vitamin C and E may lower the incidence of leukemia (12). Folate deficiency has been associated with an increased risk of liver cancer and other liver diseases (13). There is some evidence that dietary supplements containing beta carotene and vitamin E have beneficial effects, but there is also some evidence that they increase the risk of cancers such as lung and prostate (14–16). In general, further studies are required to establish a causal relationship between vitamin intake and cancer risk.

Mutations in DNA, genomic instability, and aberrant pro-tumorigenic signaling are just a few ways in which reactive oxygen species—and the myriad of internal and external variables linked to them—influence tumor development and progression (17). Diet contains a variety of essential micronutrients, including vitamins and minerals, to keep and enhance antioxidant function, influence the complex gene network (nutrigenomic approach), and encode proteins involved in carcinogenesis (18). The purpose of this study is to determine whether there is a correlation between vitamin consumption and cancer risk and enhance our knowledge of cancer prevention strategies.

## Materials and methods

### Data availability

The data used in our research came from NHANES. The NHANES aims to assess the nutritional and health status of the US population. The Health Statistics research ethics review board of the National Center has approved NHANES protocols, ensuring that they meet rigorous ethical standards.

### Study population

**Figure 1:**
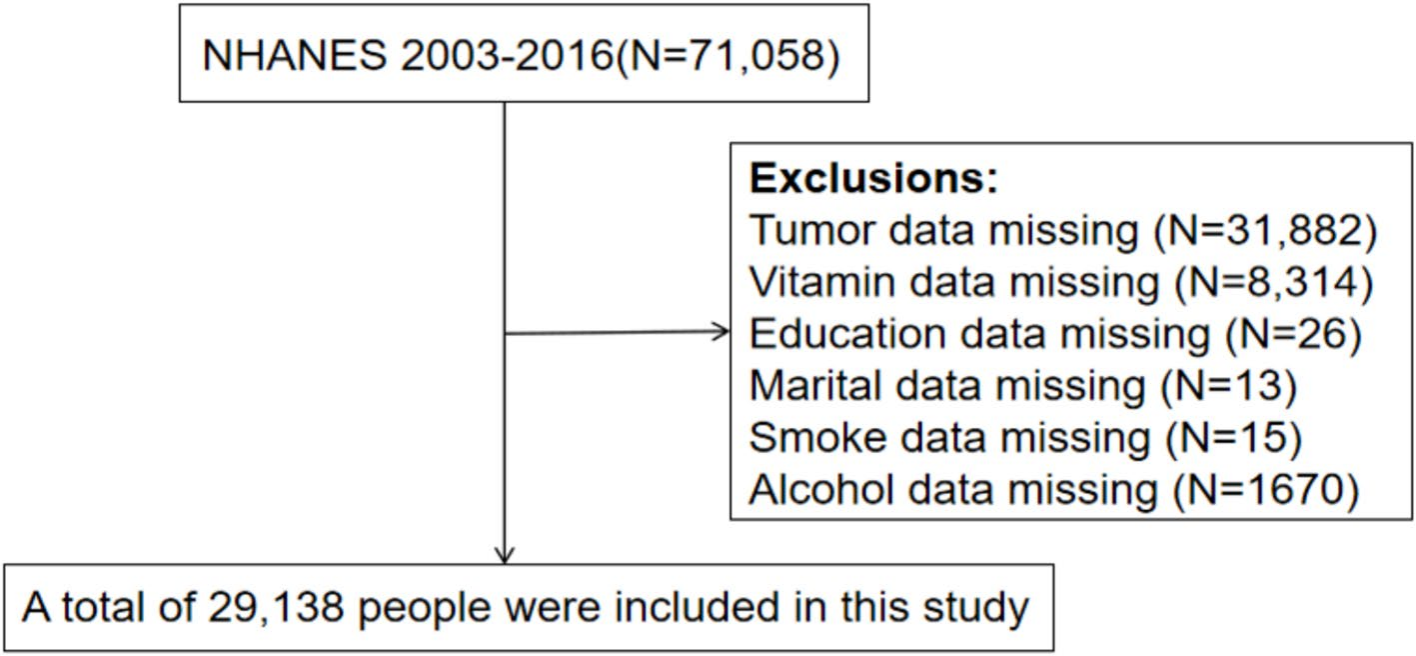
Flow chart for participants recruitment of this study.

Our analysis included seven waves of NHANES, covering the years 2003–2004, 2005–2006, 2007–2008, 2009–2010, 2011–2012, 2013–2014, and 2015–2016. The dataset included details about cancer, vitamin consumption, demographics, and personal life events. All research subjects are over 20 years old. Related studies have shown that education level and marital status are also associated with the risk of developing tumors (2, 19). Therefore, we did not include participants whose records were missing any of the following: (1) cancer (*N* = 31,882), (2) vitamin (*N* = 8,314), (3) education (*N* = 26), (4) marital status (*N* = 13), (5) smoke (*N* = 15), or (6) alcohol (*N* = 1,670). We thus included 29,138 out of 71,058 subjects (Figure 1). Specifically, we adhered to the principles outlined in the Declaration of Helsinki by the World Medical Association.

### Vitamins intake

NHANES Dietary Interview-Total Nutrient Intakes data were used. These files only include nutrient amounts that have been obtained from food and drink, including sugary drinks. They do not cover nutrients found in water, pills, or dietary supplements. Two interviews lasting 1 day each are available to all NHANES examiners regarding food recall. Two interviews are administered regarding food recall: one at the Mobile Examination Center (MEC) and 1 three to 10 days later via phone (20, 21). We used an average of the two sets of data. Regrettably, the early data did not include information on vitamin D when the data were incorporated into this study, so it was not included in this study.

### Definition of cancer

Information on a wide range of adult and pediatric health issues is available in the medical conditions section (prefix MCQ), which is an interview component. Here is how cancer is defined: Had doctor or health professional told you that you had cancer or another form of malignancy? People who said yes were considered to be part of cancerous populations.

### Covariates

We incorporated potential confounding variables, referred to as covariates, to mitigate their potential impact on the relationship between vitamin intake and malignancy risk. The selection of these covariates was informed by prior research (22, 23). Age was included as a continuous variable in our analysis. The categorical variables encompassed gender, race, educational attainment, marital status, smoking, and alcohol consumption. Smoking is the consumption of at least 100 cigarettes in one’s lifetime. There has been a period in life when the consumption of five alcoholic beverages daily for men and four for women was classified as alcohol consumption.

### Statistical analysis

Data processing and analysis were carried out using R 4.3.3 and Zstats v1.0. We performed all statistical analyses in accordance with the U.S. Center for Disease Control and Prevention guidelines. For categorical variables, we used percentages; For continuous variables, we used means and standard error. We used weighted student t-tests for continuous variables and weighted chi-square tests for categorical variables to assess the link between vitamin consumption and cancer risk. Binary logistic regression model, RCS analysis with three piecewise points were used to examine the associations between vitamin intake and cancer risk. By utilizing logistic regression analysis, subgroup analyses were carried out according to the following variables: gender, smoke, alcohol, beta-carotene, vitamin B1, vitamin B6 and folate. To evaluate the variation in associations between subgroups, we additionally utilized interaction terms. A *p*-value below 0.05 was considered statistically significant.

## Results

### Baseline characteristics of participants

Table 1 shows the demographic baseline characteristics of the study participants. A total of 29,138 people were included, with 48.13% being men and 51.87% being women. The participants’ average age was 47.40 ± 0.25 years. Respondents were divided into two groups based on population characteristics: normal and cancerous. The study’s findings revealed that tumor patients had significantly lower intakes of vitamin B1, niacin, vitamin B6, and total folate than the non-tumor population. However, tumor patients had significantly higher intakes of vitamin A, beta-carotene than the general population, with a statistically significant difference. The risk of developing a tumor was not associated with the intake of vitamins E, B2, B12, C, K and alpha-carotene. In addition, there were statistically significant differences in age, gender, race, education level, marriage and smoke between the two groups (*p* < 0.05).

**Table 1:** Analysis of the basic characteristics and differences of the research objects.

Variable	Total	Normal	Cancer	Statistic	*p*
	(*n* = 29,138)	(*n* = 26,214)	(*n* = 2,924)		
Age, Mean (SE)	47.40 (0.25)	45.62 (0.25)	62.79 (0.45)	*t* = 34.27	<0.001
Gender, *n* (%)				*χ*^2^ = 25.36	0.003
Female	15,120 (51.87)	13,580 (51.37)	1,540 (56.20)		
Male	14,018 (48.13)	12,634 (48.63)	1,384 (43.80)		
Race, *n* (%)				*χ*^2^ = 519.64	<0.001
Mexican American	4,645 (8.23)	4,479 (8.94)	166 (2.12)		
Non-Hispanic Black	6,025 (11.03)	5,636 (11.70)	389 (5.28)		
Non-Hispanic White	13,726 (69.63)	11,617 (67.57)	2,109 (87.52)		
Other Hispanic	2,447 (4.64)	2,296 (4.96)	151 (1.92)		
Other race	2,295 (6.46)	2,186 (6.84)	109 (3.17)		
Education, *n* (%)				*χ*^2^ = 30.12	<0.001
High school and below	13,885 (38.91)	12,587 (39.44)	1,298 (34.30)		
University and above	15,253 (61.09)	13,627 (60.56)	1,626 (65.70)		
Marriage, *n* (%)				*χ*^2^ = 809.82	<0.001
Divorced	3,107 (10.32)	2,736 (10.08)	371 (12.36)		
Living with partner	2,205 (7.62)	2,114 (8.11)	91 (3.35)		
Married	15,499 (55.93)	13,783 (55.07)	1716 (63.36)		
Never married	4,999 (18.13)	4,832 (19.68)	167 (4.68)		
Separated	886 (2.17)	806 (2.10)	80 (2.71)		
Widowed	2,442 (5.85)	1943 (4.96)	499 (13.54)		
Smoke, *n* (%)				*χ*^2^ = 118.57	<0.001
No	15,815 (54.25)	14,522 (55.33)	1,293 (44.91)		
Yes	13,323 (45.75)	11,692 (44.67)	1,631 (55.09)		
Alcohol, *n* (%)				*χ*^2^ = 5.63	0.097
No	8,490 (24.22)	7,590 (24.02)	900 (25.98)		
Yes	20,648 (75.78)	18,624 (75.98)	2024 (74.02)		
Vitamin E, Mean (SE)	8.23 (0.08)	8.26 (0.08)	8.02 (0.13)	*t* = −1.77	0.080
Vitamin A, Mean (SE)	650.57 (6.48)	644.15 (6.07)	706.09 (20.35)	*t* = 3.24	0.002
Alpha-carotene, Mean (SE)	421.38 (10.97)	415.34 (9.95)	473.58 (42.81)	*t* = 1.41	0.162
Beta-carotene, Mean (SE)	2254.30 (41.55)	2211.57 (39.46)	2623.72 (151.62)	*t* = 2.77	0.007
Vitamin B1, Mean (SE)	1.65 (0.01)	1.66 (0.01)	1.57 (0.02)	*t* = −4.94	<0.001
Vitamin B2, Mean (SE)	2.19 (0.01)	2.19 (0.01)	2.15 (0.03)	*t* = −1.70	0.091
Niacin, Mean (SE)	25.56 (0.13)	25.83 (0.13)	23.20 (0.24)	*t* = −10.12	<0.001
Vitamin B6, Mean (SE)	2.08 (0.01)	2.10 (0.01)	1.97 (0.02)	*t* = −4.61	<0.001
Folate, Mean (SE)	410.82 (2.84)	412.68 (2.98)	394.71 (5.25)	*t* = −3.33	0.001
Vitamin B12, Mean (SE)	5.29 (0.06)	5.31 (0.06)	5.16 (0.11)	*t* = −1.25	0.213
Vitamin C, Mean (SE)	84.38 (0.98)	84.35 (1.03)	84.67 (1.98)	*t* = 0.15	0.878
Vitamin K, Mean (SE)	111.32 (1.75)	109.76 (1.50)	124.82 (10.78)	*t* = 1.38	0.169

SE, Standard Error *t*, *t*-test, *χ*^2^, Chi-square test.

### Logistic regression analysis of vitamin intake and cancer

Vitamin A, B1, B6, beta-carotene, niacin, and total folate were all identified as independent variables with statistical significance through univariate analysis. The four quartiles were used to classify vitamin A, B1, B6, beta-carotene, niacin, and total folate intakes. Following this, we ran a logistic regression analysis. The result revealed: Niacin intake exhibited a negative correlation with tumor occurrence risk. Compared to those consuming the lowest quartile, after adjusted for gender, race, education, marriage, smoke, alcohol, age, and vitamin E, the odds of developing cancer were reduced for persons consuming the highest quartile of dietary niacin(OR = 0.76, 95%CI 0.63, 0.92, *p* = 0.005). Further adjusted for gender, race, education, marriage, smoke, alcohol, age, vitamin E, alpha-carotene, VB2, VB12, VC, VK, the risk of tumor development decreased by 22% (OR = 0.78, 95%CI 0.65, 0.95, *p* = 0.015). However, after adjusting for all confounding factors, as the intake of vitamin A gradually increased, the risk of tumor occurrence correspondingly increased (OR = 1.38, 95%CI 1.13, 1.69, *p* = 0.002). Only when the intake of folic acid is 267–367 mcg, folic acid is positively correlated with the risk of tumors. Beta-carotene, vitamin B1, vitamin B6 were not associated with the risk of tumor development (Table 2).

**Table 2:** Logistic regression analysis of vitamin intake and cancer.

Variables	Model 1	*p*	Model 2	*p*	Model 3	*p*
	OR (95% CI)		OR (95% CI)		OR (95% CI)	
Vitamin A (mcg)						
0–339	1.00 (Reference)		1.00 (Reference)		1.00 (Reference)	
340–543	1.32 (1.10 ~ 1.59)	0.004	1.09 (0.90 ~ 1.31)	0.400	1.13 (0.93 ~ 1.36)	0.213
544–819.5	1.43 (1.19 ~ 1.73)	<0.001	1.15 (0.95 ~ 1.41)	0.159	1.25 (1.02 ~ 1.53)	0.037
>819.5	1.48 (1.24 ~ 1.75)	<0.001	1.20 (1.01 ~ 1.44)	0.045	1.38 (1.13 ~ 1.69)	0.002
Beta-carotene (mcg)						
0–467.5	1.00 (Reference)		1.00 (Reference)		1.00 (Reference)	
467.6–1151.5	1.08 (0.91 ~ 1.29)	0.378	0.96 (0.81 ~ 1.14)	0.642	0.96 (0.80 ~ 1.14)	0.634
1151.6–2,809	1.54 (1.32 ~ 1.80)	<0.001	1.17 (1.00 ~ 1.38)	0.059	1.16 (0.99 ~ 1.37)	0.073
>2,809	1.40 (1.20 ~ 1.64)	<0.001	1.06 (0.89 ~ 1.25)	0.510	1.04 (0.87 ~ 1.25)	0.671
VitaminB1 (mg)						
0–1.11	1.00 (Reference)		1.00 (Reference)		1.00 (Reference)	
1.12–1.51	1.07 (0.92 ~ 1.24)	0.374	1.04 (0.88 ~ 1.21)	0.662	1.07 (0.91 ~ 1.27)	0.422
1.52–2.0	1.01 (0.88 ~ 1.18)	0.843	1.05 (0.90 ~ 1.22)	0.535	1.12 (0.95 ~ 1.32)	0.197
>2.0	0.80 (0.69 ~ 0.92)	0.002	0.97 (0.83 ~ 1.14)	0.724	1.10 (0.89 ~ 1.35)	0.403
Niacin (mg)						
0–17.29	1.00 (Reference)		1.00 (Reference)		1.00 (Reference)	
17.30–23.37	0.88 (0.77 ~ 1.02)	0.088	0.88 (0.76 ~ 1.02)	0.093	0.89 (0.77 ~ 1.03)	0.126
23.38–31.27	0.86 (0.76 ~ 0.98)	0.031	0.93 (0.81 ~ 1.06)	0.280	0.94 (0.83 ~ 1.08)	0.407
>31.27	0.57 (0.49 ~ 0.66)	<0.001	0.76 (0.63 ~ 0.92)	0.005	0.78 (0.65 ~ 0.95)	0.015
VitaminB6 (mg)						
0–1.33	1.00 (Reference)		1.00 (Reference)		1.00 (Reference)	
1.34–1.84	1.04 (0.90 ~ 1.20)	0.614	1.02 (0.87 ~ 1.19)	0.834	1.04 (0.88 ~ 1.22)	0.676
1.85–2.53	1.00 (0.86 ~ 1.17)	0.993	1.03 (0.87 ~ 1.22)	0.725	1.07 (0.89 ~ 1.27)	0.478
>2.53	0.80 (0.68 ~ 0.93)	0.005	0.97 (0.81 ~ 1.16)	0.725	1.04 (0.85 ~ 1.27)	0.698
Total Folate (mcg)						
0–266	1.00 (Reference)		1.00 (Reference)		1.00 (Reference)	
267–367	1.20 (1.03 ~ 1.39)	0.018	1.17 (1.00 ~ 1.36)	0.052	1.19 (1.01 ~ 1.39)	0.037
368–505	0.95 (0.84 ~ 1.08)	0.439	0.97 (0.83 ~ 1.13)	0.708	1.01 (0.85 ~ 1.19)	0.909
>505	0.90 (0.77 ~ 1.06)	0.209	1.07 (0.89 ~ 1.28)	0.473	1.15 (0.92 ~ 1.43)	0.214

OR, Odds Ratio; CI, Confidence Interval; Model 1, Crude; Model 2, Adjust: gender, race, education, marriage, smoke, alcohol, age, vitamin E; Model 3, Adjust: gender, race, education, marriage, smoke, alcohol, age, vitamin E, alpha-carotene, vitamin B2, vitamin B12, vitamin C, vitamin K.

### Association of vitamin and cancer

**Figure 2:**
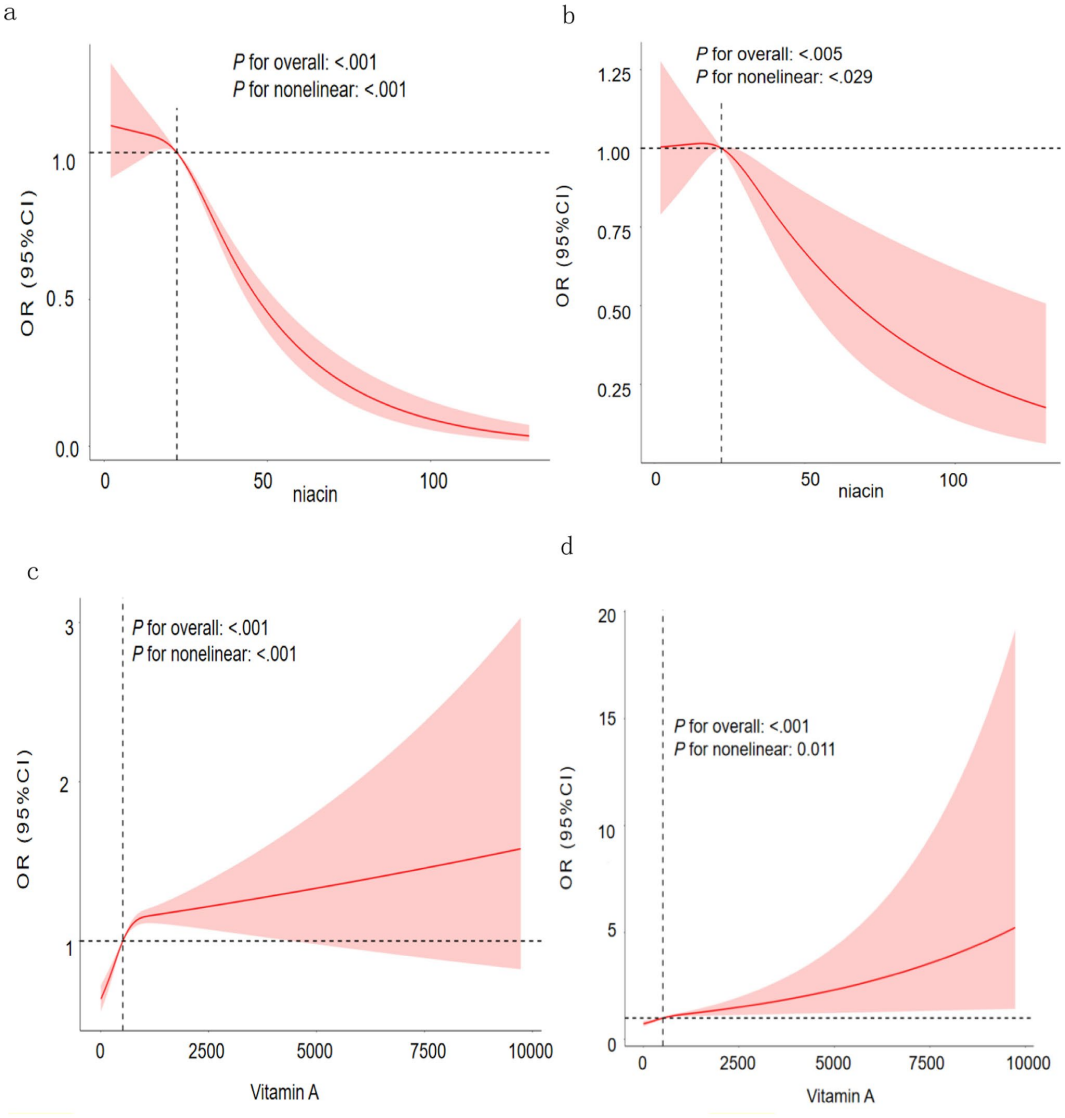
**(a)** RCS curve of the association between the niacin and cancer; **(b)** RCS curve of the association between the niacin and cancer; Adjust: age, gender, race, marriage, education, smoke, alcohol, vitamin E, vitamin A, alpha-carotene, beta-carotene, vitamin B1, vitamin B2, vitamin B6, folate, vitamin B12, vitamin C, vitamin K. **(c)** RCS curve of the association between the vitamin A and cancer; **(d)** RCS curve of the association between the vitamin A and cancer; Adjust: age, gender, race, marriage, education, smoke, alcohol, vitamin E, niacin, alpha-carotene, beta-carotene, vitamin B1, vitamin B2, vitamin B6, folate, vitamin B12, vitamin C, vitamin K.

This study employed RCS analysis to further examine the relationship between vitamin and cancer. The results indicated that niacin exhibited a negative correlation with cancer incidence in a nonlinear manner (p for nonlinear <0.05). The cancer risk diminished significantly with elevated niacin levels, particularly when exceeding 22.27 mg (Figure 2a). Further adjusted for other confounding factors, niacin intake still shows a non-linear negative correlation with the incidence of tumors (Figure 2b). Regardless of whether the mixed factor is adjusted, the vitamin A was positively correlated with the incidence of cancer and in a nonlinear pattern (p for nonlinear <0.05). Meanwhile, the risk of cancer increased slowly with the increase in vitamin A when vitamin A was greater than 511mcg (Figures 2c,d).

### Subgroup analysis

**Figure 3:**
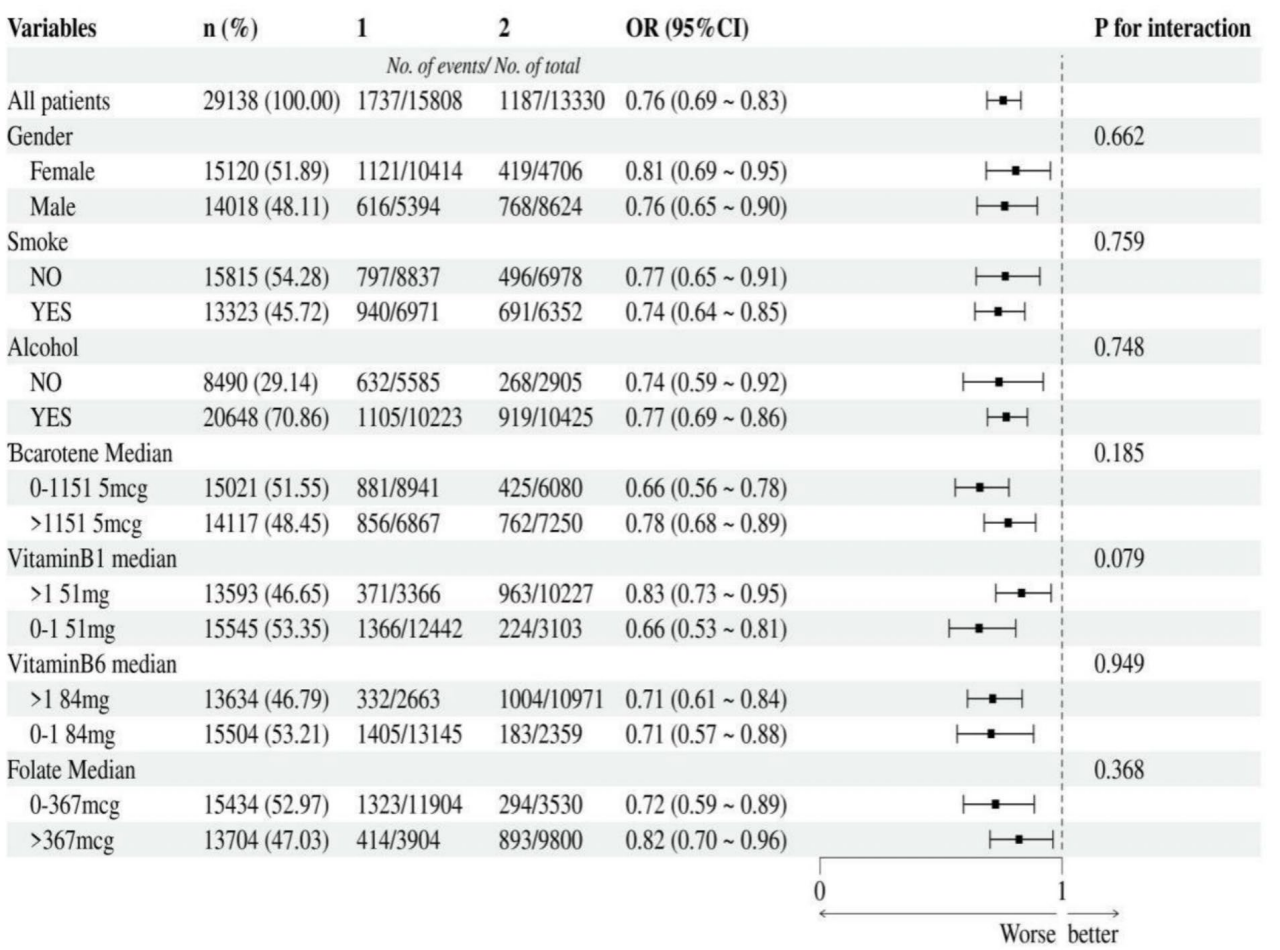
Subgroup analysis of the association between niacin intake and cancer. According to the median, niacin intake was divided into two groups: 1 = 0 to 23.37 mg, 2 was > = 23.37 mg.

**Figure 4:**
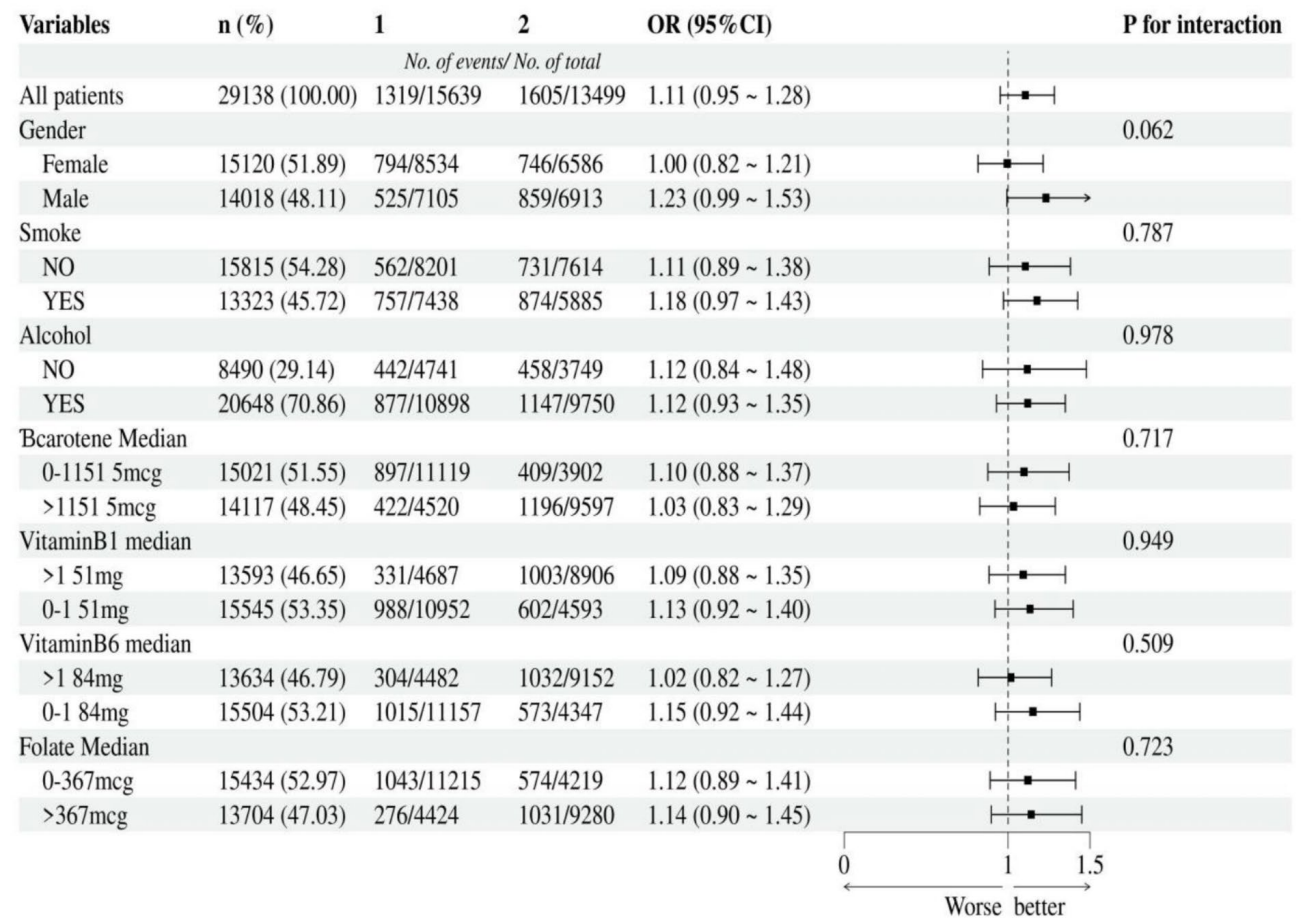
Subgroup analysis of the association between vitamin A intake and cancer. According to the median, vitamin A intake was divided into two groups: 1 = 0 to 543mcg, 2= >543mcg.

According to the median, vitamin A, B1, B6, niacin, beta-carotene, and folate were divided into two groups. The strength of the association between niacin, vitamin A intake and cancer were assessed through subgroup analysis. Additionally, we tested for significant dependence of the effect modifier on these relationship by looking for interactions with gender, smoke, alcohol, beta-carotene, vitamin B1, vitamin B6 and folate. This negative association between niacin intake and cancer remained stable in subgroups that were stratified by gender, smoke, alcohol, beta-carotene, vitamin B1, vitamin B6 and folate. And there was no correlation with the *p*-value for the interaction meeting the statistical significance (Figure 3). For the association between vitamin A intake and cancer, in subgroup stratified by gender, smoke, alcohol, beta-carotene, vitamin B1, vitamin B6 and folate, this positive association was stable. No correlation with the P for interaction meet the statistical significance was detected (Figure 4).

## Discussion

Utilizing the NHANES database, our study stands out as one of the most comprehensive cross-sectional investigations that have been conducted thus far in regards to the possible correlation between vitamin consumption and cancer in the US. While many studies have found a correlation between vitamin consumption and cancer, the exact nature of this link is still unclear. Two important findings emerged from this cross-sectional study. To start, our research shows that niacin, if consumed more often in the diet, may lower cancer risk. In addition, we found a positive correlation between vitamin A intake and cancer risk. Crucially, the correlation between vitamin A, niacin, and cancer risk persisted in multiple analyses, including logistic regression analysis, RCS and subgroup analysis.

There have been a plethora of observational studies and randomized clinical trials aimed at identifying possible dietary factors that contribute to cancer risk over the past several decades. There have been studies that have shown associations, but the results have been inconsistent or very weak (24, 25). Vitamin A derivatives influence cell proliferation, differentiation, and apoptosis, among numerous other biological processes. Loss of vision or impaired vision, night blindness, retardation, shortening and thickening of bones, impaired fetal reabsorption, and immunodeficiency are all pathological effects of vitamin A deficiency (26). According to epidemiological research, the more vitamin A you take in through your diet, the less likely you are to develop cancer (27–29). However, recent research has shown that vitamin A, which has long been believed to be an antioxidant molecule, actually has the opposite effect in living organisms. In addition, healthy subjects who use vitamin A supplements may have an increased risk of death (30). A higher category of vitamin A intake was associated with an increased risk of glioma in males, according to Giles et al. (31). Crucially, the group given retinol and beta carotene had a substantially higher risk of developing lung cancer, according to the biggest chemoprevention study on the subject to date (14). Meanwhile, study has also shown that supplementing vitamin A can increase the recurrence rate of patients with head and neck tumors (32). The differences in research on vitamin A and tumor risk can be explained by the following aspects: (1) Different study populations. For example, the research subjects in Asgari et al.’s study are mostly female, with most having a university degree or higher (14, 33, 34). (2) Different types of tumors. For instance, evidence suggests that vitamin A might elevate the risk of lung cancer and glioma in males, yet it could decrease the risk of melanoma (14, 31, 34). (3) Finally, most vitamin A - tumor risk correlation studies are retrospective case - control ones. Clinical research here has limited experimental data, the research sample size is small, and there is limited control over confounding factors (35). In contrast, our study covered adult cancer patients of all genders and all tumor types, comprehensively assessing the link between vitamin A intake and cancer risk. Using a large - sample - size database and controlling many confounding factors, we could evaluate this correlation more thoroughly. All in all, we found that vitamin A can raise the risk of malignant tumors. There is still a lack of clarity regarding the exact mechanism by which the negative effects manifest. Vitamin A has the potential to cause mitochondrial dysfunction by disrupting the homeostasis of calcium ions, vitamin A and retinoids enlarge mitochondria, which changes their structure as well (36). A cascade of effects can result from mitochondrial dysfunction. It encompasses bioenergetics deficiencies, necrosis or apoptosis, and an increase in reactive oxygen or nitrogen species production. Vitamin A may increase the risk of cancer by changing mitochondrial function and promoting electron leakage from mitochondria, which in turn increases free radical generation (8, 36). Notably, in certain experimental models, retinoids have been demonstrated to have the capacity to modify the cell cycle and trigger cell death (37). The effects of vitamin A on mitochondrial DNA damage and lipid peroxidation have been studied, vitamin A supplementation also led to redox disturbances in certain brain regions, mitochondrial membrane impairment, and respiratory chain dysfunction in rats (38). Vitamin A has the potential to enhance cancer and abnormal cell proliferation through its effects on oxidative stress, which disrupts normal intracellular signaling pathways and impacts physiological processes like cell growth, differentiation, and apoptosis. Excessive intake of Vitamin A can paradoxically increase cancer risk by promoting oxidative damage (33). In light of the fact that our findings run counter to previous research, we are concerned about the safety of dietary vitamin A supplements and whether or not they contribute to an increased risk of cancer.

Studies have shown that niacin consumption is inversely connected with the likelihood of developing carcinoma (39–43). Here is a brief rundown of the mechanisms that support this association: The anticancer effects of VB3 supplementation are first shown in a decrease in the immunosuppressive tumor microenvironment (TME) caused by myeloid cells infiltrating tumors (44). Second, nicotinamide (NAM), a niacin amide derivative that is water-soluble, initiates cell death in cancer cells by triggering ROS activation and interfering with mitochondrial function via two separate metabolic pathways (lipid metabolism and reverse electron transport) (45). The “energy crisis” in photodamaged skin is reduced because nicotinamide stimulates several protective processes in keratinocytes. This, in turn, reduces ATP depletion and increases cellular energy. In addition, nicotinamide reduces immunosuppression without lowering baseline immunity by increasing cellular energy and the activity of DNA repair enzymes (42). Lastly, our food choices greatly affect our risk of gene methylation. Niacin provides more protection against methylation than folate, according to an individual nutrient association study. It is believed that niacin can aid in DNA repair and is essential for chromosomal stability maintenance (46, 47). It is believed that micronutrient deficiencies have a major role in DNA damage, a major cancer-causing factor (48). Reduced cancer risk may be possible via niacin’s ability to strengthen the immune system, improve DNA repair, and keep genomic stability.

Our research indicates that there is no correlation between the intake of certain vitamins and an increased risk of cancer. These vitamins include E, B1, B2, B6, folate, B12, C, K, alpha-carotene and beta-carotene. Take 600 international units (IU) of vitamin E from natural sources every other day did not have any overall favorable effects on cancer. Vitamin E supplementation for cancer prevention in healthy women is not supported by this finding (49). Yang et al. concluded that vitamin E deficiency may worsen carcinogenesis and raise cancer risk, supplementation with vitamin E in this group may lower cancer risk, giving a nutrient to people who have enough of it would probably have the opposite impact (50). Vitamin B6 has conflicting effects on cancer risk; it may reduce nasopharyngeal, pancreatic, and breast cancer rates while raising hepatocellular carcinoma rates. But no significant link between B1, B2, or B12 deficiency and cancerous tumors was found (10). However, other studies have shown that an increased risk of prostate cancer has been associated with a larger daily intake of vitamin B1 and *β*-carotene, a weak inverse association between vitamin B2 consumption and breast cancer risk has been found, on top of that, taking vitamin B12 increases the likelihood of developing cancer (51–53). Researchers have found that low folate levels increase the chance of acquiring several cancers, while taking folic acid supplements or having high serum levels increases the likelihood of developing prostate cancer (54). Ascorbate deficiency may be a cause or effect of cancer, but the exact relationship is unclear. The use of vitamin C supplements did not reduce the occurrence of cancer incidence, or cancer death (55). Vitamin K helps slow the spread of some cancers, but it has also been associated with an increased risk of breast cancer (56). Overall, the relationship between vitamins and tumors is not fully understood. The complex association between vitamins and cancers is due to many variables. To start, vitamins have many different purposes, and there are many different ways in which these purposes affect the growth and presence of tumors. Second, there are huge variations throughout people when it comes to the human body. Individual differences in age, gender, genetic susceptibility, lifestyle, and medical history may moderate the effect of vitamins on cancers. Dosage effects go both ways. Too much or too little vitamin dosage can interfere with cellular metabolism and speed up tumor growth, while the right amount of vitamin promotes normal cellular processes and prevents tumor formation.

The strength of our study is the utilization of a well-documented, representative cohort to examine the correlation between vitamin intake and cancer risk. Additionally, we considered potential confounding variables that were identified in prior research, thereby minimizing confounding bias. However, it is imperative that we recognize certain constraints in our investigation. Initially, the 24-h dietary assessment may not fully capture dietary variations. Secondly, the influence of supplementary variables cannot be entirely disregarded, even after the completion of logistic regression analysis and subgroup analyses. Finally, as our data analysis uses the limited NHANES database, some covariate info is missing. For instance, details on smoking and alcohol use like quit times and durations aren’t clear. This data absence may impact research outcomes and cause confounding bias. All in all, our study explored the correlation between vitamin intake and malignant tumor occurrence via patients’ dietary vitamin intake. To some extent, it revealed this correlation. However, as a cross - sectional study, it could not fully reflect the causal relationship between vitamin intake and malignant tumor risk. Consequently, it would be advantageous to conduct longitudinal studies with a larger sample size in order to comprehend the correlation between vitamin intake and cancer risk. Our findings are medically plausible; However, they must be interpreted with prudence.

## Conclusion

Vitamin A intake is positively correlated with the occurrence of tumors, while niacin intake is negatively correlated with the incidence of tumors. Our findings demonstrated that the risk of cancer was significantly correlated with vitamin. On the other hand, in order to ascertain the precise reason behind this connection, additional extensive and long-term research is required.

## Data Availability

The raw data supporting the conclusions of this article will be made available by the authors, without undue reservation.
